# Benzyl­chloridobis(quinolin-8-olato)tin(IV)

**DOI:** 10.1107/S1600536809025902

**Published:** 2009-07-11

**Authors:** Qibao Wang

**Affiliations:** a Department of Pharmacy, Jining Medical College, No. 669 Xueyuan Road, Rizhao, Shandong 276826, People’s Republic of China

## Abstract

In the title compound, [Sn(C_7_H_7_)(C_9_H_6_NO)_2_Cl], the Sn^IV^ ion is in a distorted octa­hedral coordination environment formed by the O and N atoms of two bis-chelating quinolin-8-olate ligands, a Cl atom and a C atom from a benzyl ligand. The axial sites are occupied by an N atom of a quinolinate ligand and the C atom of the benzyl ligand. The axial Sn—N bond is slightly shorter than the equatorial Sn—N bond.

## Related literature

For the chemical, biological and pharmaceutical properties of organotin(IV) complexes, see: Nath *et al.* (2001 ▶); Pellerito & Nagy (2002 ▶). For diorganotin complexes, see: Szorcsik *et al.* (2005 ▶). For a related structure, see: Kellö *et al.* (1995 ▶).
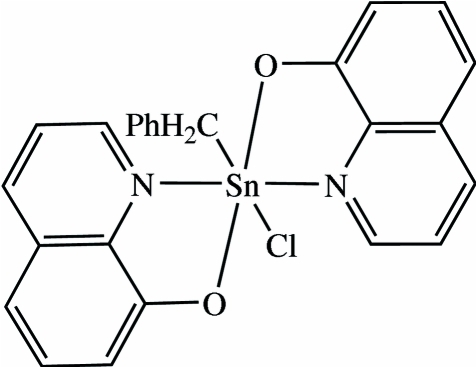


## Experimental

### 

#### Crystal data

[Sn(C_7_H_7_)(C_9_H_6_NO)_2_Cl]
*M* *_r_* = 533.56
Monoclinic, 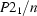

*a* = 11.6283 (14) Å
*b* = 10.6290 (14) Å
*c* = 17.948 (2) Å
β = 94.296 (2)°
*V* = 2212.1 (5) Å^3^
*Z* = 4
Mo *K*α radiation
μ = 1.30 mm^−1^
*T* = 273 K
0.20 × 0.15 × 0.12 mm

#### Data collection

Bruker APEXII diffractometer
Absorption correction: multi-scan (*SADABS*; Bruker, 2007 ▶) *T*_min_ = 0.781, *T*_max_ = 0.860
14242 measured reflections
5410 independent reflections
3930 reflections with *I* > 2σ(*I*)
*R*_int_ = 0.038

#### Refinement

*R*[*F* ^2^ > 2σ(*F* ^2^)] = 0.035
*wR*(*F* ^2^) = 0.076
*S* = 1.02
5410 reflections
281 parameters
48 restraints
H-atom parameters constrained
Δρ_max_ = 0.43 e Å^−3^
Δρ_min_ = −0.39 e Å^−3^

### 

Data collection: *APEX2* (Bruker, 2007 ▶); cell refinement: *SAINT* (Bruker, 2007 ▶); data reduction: *SAINT*; program(s) used to solve structure: *SHELXS97* (Sheldrick, 2008 ▶); program(s) used to refine structure: *SHELXL97* (Sheldrick, 2008 ▶); molecular graphics: *SHELXTL* (Sheldrick, 2008 ▶); software used to prepare material for publication: *SHELXL97*.

## Supplementary Material

Crystal structure: contains datablocks I, global. DOI: 10.1107/S1600536809025902/lh2848sup1.cif

Structure factors: contains datablocks I. DOI: 10.1107/S1600536809025902/lh2848Isup2.hkl

Additional supplementary materials:  crystallographic information; 3D view; checkCIF report

## Figures and Tables

**Table:** 

Sn1—O1	2.067 (2)
Sn1—O2	2.074 (2)
Sn1—C19	2.148 (3)
Sn1—N2	2.239 (2)
Sn1—N1	2.252 (2)
Sn1—Cl1	2.4310 (9)

**Table:** 

O1—Sn1—O2	154.94 (8)
O1—Sn1—C19	103.86 (12)
O2—Sn1—C19	95.81 (12)
O1—Sn1—N2	85.26 (8)
O2—Sn1—N2	75.96 (8)
C19—Sn1—N2	170.69 (12)
O1—Sn1—N1	76.53 (8)
O2—Sn1—N1	85.38 (8)
C19—Sn1—N1	98.59 (11)
N2—Sn1—N1	85.23 (8)
O1—Sn1—Cl1	91.98 (6)
O2—Sn1—Cl1	102.40 (6)
C19—Sn1—Cl1	93.21 (9)
N2—Sn1—Cl1	84.48 (6)
N1—Sn1—Cl1	165.16 (6)

**Fig. 1.**
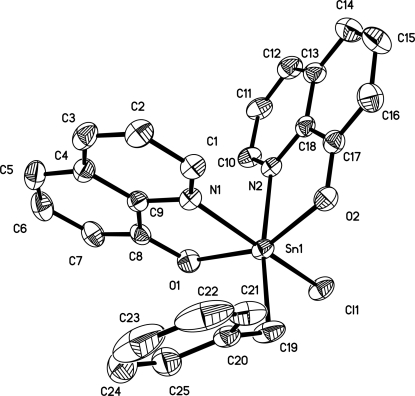
Molecular structure of title compound. Displacement ellipsoids are drawn at the 30% probability for non-H atoms.

## supplementary crystallographic information

### Comment

The chemical, biological and pharmaceutical properties of organotin(IV) complexes have been extensively studied owing to their anti-tumor activity( Pellerito & Nagy, 2002; Nath *et al.*,  2001). Organotin(IV) complexes with ligands containing phenolic –OH or phenolic –OH and –COOH groups and an aromatic N donor atom comprise an interesting class of such complexes. The studies are manily focused on diorganotin complexes (Szorcsik *et al.*,  2005) and up until now few publications have been reported on mono-organotin complexes of this type, In this paper, we reported the structure of the title mono-organotin complex. The title compound was synthesized by the reaction of sodium quinolin-8-olate with dibenzyltin dichlorides in dichloromethane. In the title compound, the coordination geometry around the Sn^IV^ ion is distorted octahedral; two O atoms, a N atom of the *cis*-chelated 8-quinolinate ligands, and a chlorine atom are in equatorial sites. The axial sites are occupied by the N atom of the other *cis*-chelate 8-quinolinate group and the C atom of the benzyl group. The two Sn—O distances are the same within experimental error but the axial Sn—N bond is slightly shorter than the equatorial Sn—N bond. The Sn—Cl and Sn—C distances are similar to those in butylchlorobis(8-quinolinate)tin(IV) (Kellö *et al.*,  1995).

### Experimental

The mixture of sodium quinolin-8-olate (0.334 g, 2.0 mmol) and dibenzyltin dichlorides (0.372 g, 1.0 mmol) was suspended in 30 ml dichloromethane at room temperature for 24 h, then the sovents were removed on a rotary evaporator, the residue was recrystallized in dichloromethane-hexane (3:1) to give yellow crystals 0.437 g. Yield 82%.

### Refinement

H atoms were positioned geometrically and refined using a riding-model approximation with C—H = 0.93–0.97 Å and with *U*_iso_(H) = 1.2 times *U*_eq_(C). The anisotropic displacement parameters of the C atoms in the benzyl group are larger than normal but were not considered severe enough to model as disorder.

### Crystal data

**Table:**

[Sn(C_7_H_7_)(C_9_H_6_NO)_2_Cl]	*F*(000) = 1064
*M**_r_* = 533.56	*D*_x_ = 1.602 Mg m^−^^3^
Monoclinic, *P*2_1_/*n*	Mo *K*α radiation, λ = 0.71073 Å
Hall symbol: -P 2yn	Cell parameters from 3464 reflections
*a* = 11.6283 (14) Å	θ = 2.2–24.1°
*b* = 10.6290 (14) Å	µ = 1.30 mm^−^^1^
*c* = 17.948 (2) Å	*T* = 273 K
β = 94.296 (2)°	Block, yellow
*V* = 2212.1 (5) Å^3^	0.20 × 0.15 × 0.12 mm
*Z* = 4	

### Data collection

**Table:**

Bruker APEXII diffractometer	5410 independent reflections
Radiation source: fine-focus sealed tube	3930 reflections with *I* > 2σ(*I*)
graphite	*R*_int_ = 0.038
φ and ω scans	θ_max_ = 28.4°, θ_min_ = 2.0°
Absorption correction: multi-scan (*SADABS*; Bruker, 2007)	*h* = −14→15
*T*_min_ = 0.781, *T*_max_ = 0.860	*k* = −14→9
14242 measured reflections	*l* = −24→23

### Refinement

**Table:**

Refinement on *F*^2^	Secondary atom site location: difference Fourier map
Least-squares matrix: full	Hydrogen site location: inferred from neighbouring sites
*R*[*F*^2^ > 2σ(*F*^2^)] = 0.035	H-atom parameters constrained
*wR*(*F*^2^) = 0.076	*w* = 1/[σ^2^(*F*_o_^2^) + (0.0307*P*)^2^] where *P* = (*F*_o_^2^ + 2*F*_c_^2^)/3
*S* = 1.02	(Δ/σ)_max_ = 0.002
5410 reflections	Δρ_max_ = 0.43 e Å^−^^3^
281 parameters	Δρ_min_ = −0.39 e Å^−^^3^
48 restraints	Extinction correction: *SHELXL97* (Sheldrick, 2008), Fc^*^=kFc[1+0.001xFc^2^λ^3^/sin(2θ)]^-1/4^
Primary atom site location: structure-invariant direct methods	Extinction coefficient: 0.00054 (14)

### Special details

**Table:**

Geometry. All e.s.d.'s (except the e.s.d. in the dihedral angle between two l.s. planes) are estimated using the full covariance matrix. The cell e.s.d.'s are taken into account individually in the estimation of e.s.d.'s in distances, angles and torsion angles; correlations between e.s.d.'s in cell parameters are only used when they are defined by crystal symmetry. An approximate (isotropic) treatment of cell e.s.d.'s is used for estimating e.s.d.'s involving l.s. planes.
Refinement. Refinement of *F*^2^ against ALL reflections. The weighted *R*-factor *wR* and goodness of fit *S* are based on *F*^2^, conventional *R*-factors *R* are based on *F*, with *F* set to zero for negative *F*^2^. The threshold expression of *F*^2^ > σ(*F*^2^) is used only for calculating *R*-factors(gt) *etc*. and is not relevant to the choice of reflections for refinement. *R*-factors based on *F*^2^ are statistically about twice as large as those based on *F*, and *R*- factors based on ALL data will be even larger.

### Fractional atomic coordinates and isotropic or equivalent isotropic displacement parameters (Å^2^)

**Table:**

	*x*	*y*	*z*	*U*_iso_*/*U*_eq_	
Sn1	0.695432 (15)	0.868135 (18)	0.124643 (11)	0.03912 (8)	
Cl1	0.65912 (7)	1.09309 (8)	0.12976 (5)	0.0555 (2)	
N1	0.69034 (19)	0.6613 (2)	0.09745 (13)	0.0384 (5)	
N2	0.50410 (19)	0.8508 (2)	0.12869 (13)	0.0376 (5)	
O1	0.66194 (18)	0.86474 (18)	0.00990 (11)	0.0476 (5)	
O2	0.67424 (16)	0.8119 (2)	0.23340 (10)	0.0457 (5)	
C1	0.7089 (2)	0.5632 (3)	0.14258 (17)	0.0436 (7)	
H1	0.7284	0.5771	0.1931	0.052*	
C2	0.7000 (2)	0.4408 (3)	0.11637 (18)	0.0529 (8)	
H2	0.7139	0.3734	0.1489	0.064*	
C3	0.6711 (3)	0.4201 (3)	0.04313 (19)	0.0544 (8)	
H3	0.6646	0.3378	0.0256	0.065*	
C4	0.6504 (2)	0.5201 (3)	−0.00712 (17)	0.0441 (7)	
C5	0.6201 (3)	0.5090 (4)	−0.08445 (19)	0.0595 (9)	
H5	0.6123	0.4300	−0.1066	0.071*	
C6	0.6023 (3)	0.6147 (4)	−0.12630 (19)	0.0626 (10)	
H6	0.5814	0.6065	−0.1771	0.075*	
C7	0.6145 (3)	0.7350 (4)	−0.09581 (17)	0.0537 (8)	
H7	0.6003	0.8048	−0.1264	0.064*	
C8	0.6468 (2)	0.7521 (3)	−0.02160 (16)	0.0419 (7)	
C9	0.6630 (2)	0.6424 (3)	0.02335 (15)	0.0374 (6)	
C10	0.4226 (2)	0.8810 (3)	0.07682 (17)	0.0454 (7)	
H10	0.4433	0.9129	0.0315	0.055*	
C11	0.3059 (3)	0.8664 (3)	0.0881 (2)	0.0553 (9)	
H11	0.2498	0.8914	0.0515	0.066*	
C12	0.2751 (3)	0.8152 (3)	0.1531 (2)	0.0587 (9)	
H12	0.1975	0.8036	0.1606	0.070*	
C13	0.3607 (2)	0.7794 (3)	0.20950 (18)	0.0479 (7)	
C14	0.3386 (3)	0.7245 (4)	0.2775 (2)	0.0685 (10)	
H14	0.2634	0.7056	0.2879	0.082*	
C15	0.4281 (4)	0.6989 (4)	0.3284 (2)	0.0752 (11)	
H15	0.4126	0.6614	0.3734	0.090*	
C16	0.5433 (3)	0.7270 (3)	0.31570 (18)	0.0595 (9)	
H16	0.6021	0.7095	0.3522	0.071*	
C17	0.5684 (2)	0.7800 (3)	0.24933 (16)	0.0428 (7)	
C18	0.4754 (2)	0.8027 (3)	0.19468 (16)	0.0388 (6)	
C19	0.8792 (2)	0.8914 (3)	0.1398 (2)	0.0616 (10)	
H19A	0.9022	0.9534	0.1042	0.074*	
H19B	0.8983	0.9249	0.1894	0.074*	
C20	0.9480 (2)	0.7754 (3)	0.13088 (17)	0.0453 (7)	
C21	0.9725 (3)	0.6970 (4)	0.1901 (2)	0.0668 (10)	
H21	0.9468	0.7171	0.2364	0.080*	
C22	1.0347 (4)	0.5888 (5)	0.1821 (4)	0.115 (2)	
H22	1.0519	0.5367	0.2231	0.138*	
C23	1.0711 (5)	0.5578 (6)	0.1143 (5)	0.141 (3)	
H23	1.1122	0.4838	0.1086	0.170*	
C24	1.0482 (4)	0.6336 (6)	0.0557 (4)	0.118 (2)	
H24	1.0742	0.6126	0.0096	0.142*	
C25	0.9858 (3)	0.7432 (4)	0.0632 (2)	0.0732 (11)	
H25	0.9697	0.7951	0.0221	0.088*	

### Atomic displacement parameters (Å^2^)

**Table:**

	*U*^11^	*U*^22^	*U*^33^	*U*^12^	*U*^13^	*U*^23^
Sn1	0.03405 (11)	0.03303 (12)	0.04976 (14)	−0.00005 (9)	−0.00043 (8)	−0.00080 (9)
Cl1	0.0532 (4)	0.0332 (4)	0.0790 (6)	0.0005 (3)	−0.0019 (4)	−0.0058 (4)
N1	0.0368 (12)	0.0351 (14)	0.0427 (13)	0.0014 (10)	−0.0021 (10)	−0.0002 (11)
N2	0.0352 (12)	0.0321 (13)	0.0449 (13)	−0.0018 (10)	−0.0021 (10)	−0.0019 (11)
O1	0.0589 (13)	0.0367 (12)	0.0478 (12)	0.0030 (10)	0.0069 (10)	0.0074 (10)
O2	0.0413 (11)	0.0489 (13)	0.0454 (11)	0.0001 (10)	−0.0066 (9)	0.0001 (10)
C1	0.0415 (16)	0.0403 (18)	0.0482 (17)	0.0036 (14)	−0.0016 (13)	0.0021 (14)
C2	0.0550 (19)	0.0406 (18)	0.062 (2)	0.0063 (15)	−0.0037 (16)	0.0055 (16)
C3	0.0527 (18)	0.0365 (18)	0.073 (2)	0.0043 (15)	0.0001 (17)	−0.0106 (17)
C4	0.0350 (14)	0.0447 (18)	0.0523 (18)	0.0044 (13)	0.0012 (13)	−0.0083 (15)
C5	0.059 (2)	0.057 (2)	0.062 (2)	0.0075 (17)	−0.0002 (17)	−0.0210 (18)
C6	0.062 (2)	0.084 (3)	0.0419 (17)	0.013 (2)	0.0022 (15)	−0.0081 (19)
C7	0.056 (2)	0.061 (2)	0.0444 (18)	0.0119 (17)	0.0076 (15)	0.0053 (17)
C8	0.0364 (15)	0.0445 (19)	0.0461 (17)	0.0055 (13)	0.0109 (13)	−0.0015 (14)
C9	0.0315 (13)	0.0369 (16)	0.0440 (16)	0.0036 (12)	0.0042 (12)	−0.0011 (13)
C10	0.0404 (15)	0.0417 (18)	0.0526 (18)	−0.0022 (13)	−0.0063 (13)	0.0058 (14)
C11	0.0379 (16)	0.056 (2)	0.070 (2)	−0.0025 (15)	−0.0126 (15)	0.0040 (18)
C12	0.0385 (17)	0.058 (2)	0.080 (2)	−0.0019 (16)	0.0044 (17)	−0.0098 (19)
C13	0.0395 (16)	0.0464 (19)	0.0586 (19)	−0.0030 (14)	0.0090 (14)	−0.0059 (16)
C14	0.060 (2)	0.081 (3)	0.066 (2)	−0.011 (2)	0.0183 (19)	0.001 (2)
C15	0.088 (3)	0.085 (3)	0.056 (2)	−0.009 (2)	0.024 (2)	0.013 (2)
C16	0.066 (2)	0.065 (2)	0.0467 (19)	0.0048 (18)	−0.0019 (17)	0.0064 (18)
C17	0.0430 (17)	0.0391 (17)	0.0462 (17)	0.0024 (13)	0.0022 (13)	−0.0048 (14)
C18	0.0417 (15)	0.0298 (16)	0.0450 (17)	0.0017 (12)	0.0042 (13)	−0.0062 (13)
C19	0.0335 (16)	0.050 (2)	0.101 (3)	−0.0059 (15)	−0.0019 (17)	−0.0011 (19)
C20	0.0289 (14)	0.0435 (18)	0.063 (2)	−0.0024 (13)	−0.0001 (14)	−0.0020 (16)
C21	0.0453 (19)	0.068 (3)	0.086 (3)	−0.0144 (17)	−0.0035 (18)	0.019 (2)
C22	0.060 (3)	0.063 (3)	0.215 (6)	−0.011 (2)	−0.030 (3)	0.053 (4)
C23	0.060 (3)	0.058 (3)	0.304 (9)	0.005 (3)	−0.001 (5)	−0.045 (4)
C24	0.061 (3)	0.130 (5)	0.167 (5)	−0.014 (3)	0.034 (3)	−0.089 (4)
C25	0.050 (2)	0.101 (3)	0.069 (2)	−0.007 (2)	0.0030 (18)	−0.015 (2)

### Geometric parameters (Å, °)

**Table:**

Sn1—O1	2.067 (2)	C10—H10	0.9300
Sn1—O2	2.074 (2)	C11—C12	1.359 (5)
Sn1—C19	2.148 (3)	C11—H11	0.9300
Sn1—N2	2.239 (2)	C12—C13	1.418 (4)
Sn1—N1	2.252 (2)	C12—H12	0.9300
Sn1—Cl1	2.4310 (9)	C13—C14	1.393 (4)
N1—C1	1.328 (4)	C13—C18	1.401 (4)
N1—C9	1.359 (3)	C14—C15	1.360 (5)
N2—C10	1.318 (3)	C14—H14	0.9300
N2—C18	1.355 (3)	C15—C16	1.408 (5)
O1—C8	1.330 (3)	C15—H15	0.9300
O2—C17	1.328 (3)	C16—C17	1.369 (4)
C1—C2	1.385 (4)	C16—H16	0.9300
C1—H1	0.9300	C17—C18	1.425 (4)
C2—C3	1.350 (4)	C19—C20	1.485 (4)
C2—H2	0.9300	C19—H19A	0.9700
C3—C4	1.403 (4)	C19—H19B	0.9700
C3—H3	0.9300	C20—C21	1.364 (4)
C4—C5	1.411 (4)	C20—C25	1.366 (4)
C4—C9	1.414 (4)	C21—C22	1.371 (6)
C5—C6	1.359 (5)	C21—H21	0.9300
C5—H5	0.9300	C22—C23	1.359 (8)
C6—C7	1.394 (5)	C22—H22	0.9300
C6—H6	0.9300	C23—C24	1.335 (9)
C7—C8	1.369 (4)	C23—H23	0.9300
C7—H7	0.9300	C24—C25	1.384 (7)
C8—C9	1.422 (4)	C24—H24	0.9300
C10—C11	1.395 (4)	C25—H25	0.9300
			
O1—Sn1—O2	154.94 (8)	N2—C10—H10	119.1
O1—Sn1—C19	103.86 (12)	C11—C10—H10	119.1
O2—Sn1—C19	95.81 (12)	C12—C11—C10	119.4 (3)
O1—Sn1—N2	85.26 (8)	C12—C11—H11	120.3
O2—Sn1—N2	75.96 (8)	C10—C11—H11	120.3
C19—Sn1—N2	170.69 (12)	C11—C12—C13	120.3 (3)
O1—Sn1—N1	76.53 (8)	C11—C12—H12	119.9
O2—Sn1—N1	85.38 (8)	C13—C12—H12	119.9
C19—Sn1—N1	98.59 (11)	C14—C13—C18	118.7 (3)
N2—Sn1—N1	85.23 (8)	C14—C13—C12	124.9 (3)
O1—Sn1—Cl1	91.98 (6)	C18—C13—C12	116.4 (3)
O2—Sn1—Cl1	102.40 (6)	C15—C14—C13	119.4 (3)
C19—Sn1—Cl1	93.21 (9)	C15—C14—H14	120.3
N2—Sn1—Cl1	84.48 (6)	C13—C14—H14	120.3
N1—Sn1—Cl1	165.16 (6)	C14—C15—C16	122.6 (3)
C1—N1—C9	119.8 (3)	C14—C15—H15	118.7
C1—N1—Sn1	129.3 (2)	C16—C15—H15	118.7
C9—N1—Sn1	110.89 (18)	C17—C16—C15	119.7 (3)
C10—N2—C18	119.9 (2)	C17—C16—H16	120.1
C10—N2—Sn1	128.4 (2)	C15—C16—H16	120.1
C18—N2—Sn1	111.70 (17)	O2—C17—C16	123.6 (3)
C8—O1—Sn1	116.63 (18)	O2—C17—C18	118.4 (3)
C17—O2—Sn1	116.97 (17)	C16—C17—C18	117.9 (3)
N1—C1—C2	121.7 (3)	N2—C18—C13	122.2 (3)
N1—C1—H1	119.1	N2—C18—C17	116.2 (2)
C2—C1—H1	119.1	C13—C18—C17	121.6 (3)
C3—C2—C1	119.4 (3)	C20—C19—Sn1	115.5 (2)
C3—C2—H2	120.3	C20—C19—H19A	108.4
C1—C2—H2	120.3	Sn1—C19—H19A	108.4
C2—C3—C4	121.4 (3)	C20—C19—H19B	108.4
C2—C3—H3	119.3	Sn1—C19—H19B	108.4
C4—C3—H3	119.3	H19A—C19—H19B	107.5
C3—C4—C5	125.9 (3)	C21—C20—C25	118.5 (4)
C3—C4—C9	116.1 (3)	C21—C20—C19	120.4 (3)
C5—C4—C9	117.9 (3)	C25—C20—C19	121.1 (3)
C6—C5—C4	119.4 (3)	C20—C21—C22	120.8 (4)
C6—C5—H5	120.3	C20—C21—H21	119.6
C4—C5—H5	120.3	C22—C21—H21	119.6
C5—C6—C7	122.3 (3)	C23—C22—C21	119.9 (6)
C5—C6—H6	118.8	C23—C22—H22	120.0
C7—C6—H6	118.8	C21—C22—H22	120.0
C8—C7—C6	121.1 (3)	C24—C23—C22	120.1 (6)
C8—C7—H7	119.5	C24—C23—H23	120.0
C6—C7—H7	119.5	C22—C23—H23	120.0
O1—C8—C7	123.5 (3)	C23—C24—C25	120.4 (6)
O1—C8—C9	119.3 (3)	C23—C24—H24	119.8
C7—C8—C9	117.3 (3)	C25—C24—H24	119.8
N1—C9—C4	121.6 (3)	C20—C25—C24	120.2 (5)
N1—C9—C8	116.4 (3)	C20—C25—H25	119.9
C4—C9—C8	121.9 (3)	C24—C25—H25	119.9
N2—C10—C11	121.7 (3)		
